# Promoting the Use of Common Oat Genetic Resources through Diversity Analysis and Core Collection Construction

**DOI:** 10.1371/journal.pone.0167855

**Published:** 2016-12-13

**Authors:** Maja Boczkowska, Bogusław Łapiński, Izabela Kordulasińska, Denise F. Dostatny, Jerzy H. Czembor

**Affiliations:** National Centre for Plant Genetic Resources, Plant Breeding and Acclimatization Institute (IHAR) - National Research Institute, Radzików, Poland; USDA-ARS Southern Regional Research Center, UNITED STATES

## Abstract

The assessment of diversity and population structure and construction of a core collection is beneficial for the efficient use and management of germplasm. A unique collection of common oat landraces, cultivated in the temperate climate of central Europe until the end of the twentieth century, is preserved in the Polish gene bank. It consists of 91 accessions that have never been used in breeding programs. In order to optimise the use of this genetic resource, we aimed to: (1) determine genetic and agro-morphological diversity, (2) identify internal genetic variation of the tested accessions, (3) form a core collection and (4) recognise the accessions useful for breeding programs or re-release for cultivation. The collection was screened using ISSR markers (1520 loci) and eight agro-morphological traits. Uniquely, we performed molecular studies based on 24 individuals of every accession instead of bulk samples. Therefore, assessment of the degree of diversity within each population and the identification of overlapping gene pools were possible. The observed internal diversity (Nei unbiased coefficient) was in the range of 0.17–0.31. Based on combined genetic and agro-morphological data, we established the core collection composed of 21 landraces. Due to valuable compositions of important traits, some accessions were also identified as useful for breeding programs. The population structure and principal coordinate analysis revealed two major clusters. Based on the previous results, the accessions classified within the smaller one were identified as obsolete varieties instead of landraces. Our results show that the oat landraces are, in general, resistant to local races of diseases, well adapted to local conditions and, in some cases, yielding at the level of modern varieties. Therefore, *in situ* conservation of the landraces in the near future may be satisfactory for both farmers and researchers in terms of the genetic resources preservation.

## Introduction

The domestication of plants can be considered as one of the greatest achievements of mankind. It is associated with the long-term and targeted selection which greatly reduced the gene pool size of the domesticated species. It is estimated that the crop species retained only about 20% of the original variability of their wild ancestors [1–3]. Moreover, modern crop varieties are almost or completely homogeneous and closely related to each other, which further narrows the exploited gene pool [4]. Modern breeding relying on the subsequent crossing of descendants of several initial germplasms has led to the loss of numerous alleles which, in turn, limited the potential of phenotype and genotype pools that could be useful for further breeding. Monocultures of homogeneous varieties are considered to be the main reason for breaking down the plant resistance by pathogens and, consequently, for outbreaks of epidemics such as the Southern Corn Leaf Blight caused by race T of the fungus *Bipolaris maydis* in the 1970’s in the United States [5]. The lack of new germplasm for breeding has increased the interest in wild crop relatives (CWR) as new sources of genes for further crop improvement. However, the use of alien germplasm often involves a large amount of work due to the need to eliminate the useless or even detrimental genetic background or to overcome reproductive isolation barriers [6]. Landraces represent an easier to use source of lost traits/alleles than wild species. They are the connecting link between modern varieties or breeding stocks and wild ancestors. The domestication was a long-term process and resulted in the development of landraces adapted to local farmers’ needs and environmental conditions. According to the definition, landraces are heterogeneous populations that were not obtained by deliberate crop improvement, but were formed by mutations and migrations under selection pressure of biotic and abiotic stresses and by the needs and behaviour of generations of farmers [7]. Thousands of landraces are stored in gene banks worldwide; however, the majority of them are inadequately described, which impedes or even prevents their effective use in breeding programs. The breeders deliberately refrain from the use of landraces due to the high costs and time-lapse of extensive search [8]. Intensive pre-breeding approaches are essential for promoting the transfer of specific genes or traits from unimproved landraces to advanced breeding lines [9].

Despite the development of the next-generation sequencing, the DNA fingerprinting is still the preferred way of plant genetic resources (PGR) genetic characterization [10–13]. Molecular biology provides various methods based on the DNA polymorphism which are not affected by the environment; they require only a small amounts of biological material at any growth stage that can be easily transported from the collection site to a laboratory and stored for a long time [14]. There is a potentially unlimited number of molecular markers which cover almost the entire genome [15]. In genetic diversity studies, many different types of molecular markers have been used, including RFLP (Restriction Fragments Length Polymorphism), RAPD (Random Amplified Polymorphic DNA), AFLP (Amplified Fragment Length Polymorphism), ISSR (Inter-Simple Sequence Repeats), SSR (Simple Sequence Repeats), CAPS (Cleaved Amplified Polymorphic Sequences), SNP (Single Nucleotide Polymorphism), etc. [10,16–18]. ISSR is cheap and effective genotyping technique that have been used for determining genetic diversity [19,20]. ISSRs detect variations of DNA fragments located between two adjacent, identical and opposite orientated microsatellites [21]. The prior knowledge of DNA sequences is not required to design PCR primers. ISSRs are dominant markers i.e. they do not distinguish between homo- and heterozygotes at a given locus [22]. Although during the last two decades, molecular analysis for genetic diversity estimation has become more common and more cost effective, the amount of this type of data used for accession description has not increased as fast as it was anticipated in 1996 [23].

The overall aims of this work were (i) to determine ISSR based genetic diversity of the oat landraces stored in the Polish gene bank, (ii) to evaluate variation of agro-morphological traits, (iii) to identify internal genetic variation of each tested accession, (iv) to develop a core set representing the maximum diversity of Polish oat landraces and (v) to recognise accessions potentially useful for breeding programs or re-release for cultivation.

## Materials and Methods

### Plant material

The study involved 91 indigenous accessions of common oat (Table 1) preserved by the National Centre for Plant Genetic Resources in Radzików, i.e. the Polish gene bank. In this set, the whole collection of Polish oat landraces was included, as well as three heterogeneous accessions which were identified earlier as obsolete varieties i.e. PL 50076 _(1)_, PL 50079 _(2)_ and PL 50381 _(5)_ [24]. Each of the tested accessions was represented by 24 individuals which were analysed separately.

**Table 1:** Accession list containing some basic information.

No.	Accession no.	Latitude	Longitude	uHe		ISSR data	Evaluation data	Core collection
				Mean	SE			
1	PL 50076	*na*	*na*	0.231	0.007	+	+	
2	PL 50079	4924--N	02021--E	0.171	0.009	+	+	+
3	PL 50338	4931--N	02209--E	0.24	0.008	+	+	
4	PL 50345	5018--N	02145--E	0.241	0.009	+	+	
5	PL 50381	5018--N	02145--E	0.251	0.008	+	+	+
6	PL 50411	5027--N	02317--E	0.195	0.006	+	+	
7	PL 50432	4924--N	02018--E	0.205	0.007	+	+	+
8	PL 50438	5040--N	02310--E	0.237	0.007	+	+	
9	PL 50503	5044--N	02315--E	0.225	0.009	+	+	+
10	PL 50512	4930--N	02014--E	0.22	0.007	+	+	
11	PL 50520	5216--N	02219--E	0.196	0.008	+	+	
12	PL 50521	4933--N	02059--E	0.256	0.008	+	+	
13	PL 50524	4932--N	02206--E	0.238	0.007	+	+	
14	PL 50528	4929--N	02216--E	0.244	0.007	+	+	
15	PL 50529	5338--N	02309--E	0.213	0.007	+	+	+
16	PL 50530	5044--N	02315--E	0.211	0.007	+	+	
17	PL 50531	5301--N	02321--E	0.217	0.007	+	+	
18	PL 50553	4930--N	02205--E	0.235	0.007	+	+	
19	PL 50554	4932--N	02102--E	0.24	0.007	+	+	
20	PL 50556	4930--N	02014--E	0.3	0.005	+	+	
21	PL 50561	5216--N	02219--E	0.288	0.006	+	+	
22	PL 50587	5115--N	02234--E	0.255	0.006	+	+	
23	PL 50593	4930--N	02014--E	0.249	0.007	+	+	
24	PL 50613	5039--N	02322--E	0.234	0.009	+	+	+
25	PL 50616	4935--N	02028--E	0.212	0.007	+	+	
26	PL 50622	4936--N	02225--E	0.231	0.008	+	+	
27	PL 50627	5215--N	02153--E	0.234	0.007	+	+	
28	PL 50673	5033--N	02336--E	0.219	0.007	+	+	
29	PL 50694	4927--N	02220--E	0.229	0.007	+	+	
30	PL 50698	5049--N	02219--E	0.227	0.007	+	+	
31	PL 50705	4924--N	02018--E	0.202	0.007	+	+	
32	PL 50706	4924--N	02018--E	0.194	0.007	+	+	
33	PL 50709	4948--N	02154--E	0.254	0.007	+	+	
34	PL 50712	5022--N	02150--E	0.196	0.007	+	+	
35	PL 50718	5102--N	02300--E	0.189	0.007	+	+	
36	PL 50725	4923--N	02008--E	0.268	0.006	+	+	
37	PL 50754	4930--N	02030--E	0.222	0.007	+	+	
38	PL 50758	5420--N	02256--E	0.237	0.007	+	+	
39	PL 50760	5039--N	02316--E	0.206	0.008	+	+	
40	PL 50902	4922--N	02011--E	0.223	0.007	+	+	
41	PL 50904	4922--N	02011--E	0.231	0.007	+	+	
42	PL 50925	4930--N	02024--E	0.218	0.007	+	+	+
43	PL 50945	4922--N	02011--E	0.218	0.007	+	+	
44	PL 51439	*na*	*na*	0.313	0.008	+	+	+
45	PL 51440	*na*	*na*	0.249	0.006	+	+	+
46	PL 51441	*na*	*na*	0.269	0.006	+	+	
47	PL 51442	*na*	*na*	0.299	0.009	+	+	
48	PL 51443	*na*	*na*	0.241	0.009	+	+	
49	PL 51519	4922--N	02011--E	0.231	0.007	+	+	
50	PL 51521	5150--N	02229--E	0.277	0.007	+	+	
51	PL 51522	5107--N	02320--E	0.252	0.007	+	+	
52	PL 51599	5052--N	02304--E	0.225	0.007	+	+	
53	PL 51600	5010--N	02236--E	0.211	0.008	+	+	
54	PL 51603	50----N	02240--E	0.25	0.007	+	+	
55	PL 51604	4941--N	02229--E	0.231	0.007	+	+	+
56	PL 51605	4941--N	02231--E	0.231	0.007	+	+	
57	PL 51606	5224--N	01947--E	0.2	0.007	+	+	
58	PL 51607	5000--N	02235--E	0.182	0.007	+	+	
59	PL 51609	5048--N	02258--E	0.242	0.008	+	+	
60	PL 51610	5003--N	02234--E	0.222	0.007	+	+	
61	PL 51611	5001--N	02238--E	0.254	0.007	+	+	
62	PL 51612	5032--N	02336--E	0.228	0.007	+	+	
63	PL 51614	5037--N	02259--E	0.243	0.007	+	+	
64	PL 51615	5051--N	02326--E	0.201	0.007	+	+	+
65	PL 51616	4921--N	02012--E	0.272	0.006	+	+	
66	PL 51617	4922--N	02011--E	0.215	0.007	+	+	
67	PL 51618	4922--N	02001--E	0.265	0.006	+	+	+
68	PL 51619	4922--N	02011--E	0.221	0.007	+	+	
69	PL 51632	*na*	*na*	0.208	0.006	+	+	+
70	PL 51634	*na*	*na*	0.199	0.008	+	*na*	+
71	PL 51687	*na*	*na*	0.236	0.007	+	+	
72	PL 52189	*na*	*na*	0.232	0.007	+	*na*	
73	PL 52191	4921--N	02012--E	0.263	0.006	+	+	
74	PL 52193	*na*	*na*	0.236	0.007	+	+	+
75	PL 52194	*na*	*na*	0.264	0.01	+	+	+
76	PL 52195	*na*	*na*	0.269	0.008	+	+	
77	PL 52255	5158--N	02224--E	0.199	0.008	+	+	+
78	PL 52265	4945--N	01916--E	0.272	0.007	+	+	
79	PL 52267	*na*	*na*	0.203	0.007	+	*na*	
80	PL 52268	5318--N	02200--E	0.229	0.008	+	+	
81	PL 52306	5048--N	02314--E	0.255	0.007	+	+	
82	PL 52338	5026--N	02026--E	0.192	0.007	+	+	
83	PL 52343	5254--N	02252--E	0.232	0.008	+	+	
84	PL 52344	4930--N	01945--E	0.21	0.008	+	+	+
85	PL 52351	5301--N	02250--E	0.287	0.006	+	+	+
86	PL 52429	5314--N	02135--E	0.166	0.008	+	*na*	
87	PL 52430	*na*	*na*	0.211	0.007	+	*na*	+
88	PL 52431	*na*	*na*	0.277	0.007	+	*na*	
89	PL 52432	*na*	*na*	0.3	0.008	+	*na*	
90	PL 52433	*na*	*na*	0.262	0.006	+	+	
91	PL 52434	*na*	*na*	0.225	0.009	+	+	+

[uHe—the unbiased Nei coefficient for intra-accession genetic diversity; *na*–not available]

### Agro-morphological traits

Agro-morphological data were obtained from the EGISET i.e. the database of NCPGR [25]. Evaluation was conducted on the basis of field experiments during three subsequent seasons, using standardised scales and descriptors. The trial was carried out on an experimental field of the Plant Breeding and Acclimatization Institute at Radzików, Poland in 1981–2012. Each accession was represented by 1000 grains which were sown on the 2.5 m^2^ plots. The characters were scored at an appropriate growth stage of the oat plants following the International Convention for the Protection of New Varieties of Plants [26]. We analysed the following eight traits: the number of days to heading, the number of days to maturity, the shoot height, the lodging score at mature stage, the susceptibility to diseases (crown rust, powdery mildew), the grain yield and the thousand seed weight. The number of days to heading was counted from the sowing date to 50% of panicles fully emerged. The number of days to maturity was counted from the sowing date to harvest ripeness. The shoot height was determined by the average of three plants measured from ground to the tip of panicle (excluding awns). The lodging score was determined in mature stage according to a scale where: 1 means all plants lodged and 9 no lodging at all. The susceptibility to diseases was also determined according to a scale where: 1 means affected and 9 means resistant (based on percentage of affected plants). Both diseases were evaluated separately. The grain yield was assessed as the weight of grain per plot. The thousand seed weight was estimated based on the average weight of three samples of 100 fully filled grains.

### ISSR genotyping

The DNA of 2,184 individual plantlets was extracted from tissue of young leaves using the Genomic Mini AX Plant kit (A&A Biotechnology). The DNA concentration was determined spectrophotometrically (NanoDrop Sectrophotometer ND-1000) and the DNA samples were diluted to 25 ng μl^−1^ for PCR amplification.

The previously described set of eight ISSR primers [27] was applied to the studies on diversity of Polish oat landraces. The PCR was carried out according to the protocol described by Boczkowska et al. [28]. The multiplexed amplified PCR products were separated on the capillary sequencer ABI 3130 XL Genetic Analyzer in a 36-cm long capillary array filled with POP7 polymer. Gene Scan 1200 LIZ (Applied Biosystem) was used as a size standard. The genotypic data are available on the EGISET database.

### Scoring and data analysis

The agro-morphological data were standardised and a proximity matrix by the Gower coefficient [29] was developed. Subsequently, we performed the Ward’s independent, agglomerative hierarchical clustering procedure and plotted the resulting dendrogram. The principal components analysis (PCA) was applied to the initially standardised data and the 2-D scatter plots were generated. The phenotypic frequency data of the eight characteristics were analysed by the Shannon-Weaver diversity index, H, defined as:

$$H=-\sum_{i}^{k}\left(p_{i}lnp_{i}\right)$$

where *k* is the number of phenotypic classes for a character and *p*_*i*_ is the proportion of the total number of entries in the i^th^ class [30]. For this, the overall phenotype range was divided into 12 phenotype classes of similar size.

In addition, we calculated the Pearson correlation coefficients between all agro-morphological traits (p < 0.05).

Initially, the GeneMapper software (Applied Biosystem) was used to analyse the capillary electrophoresis raw data. A binary matrix of the ISSR results was developed in the MS Excel 2010. Only clear and apparently unambiguous peaks were scored. The informativeness of a marker was determined by the Polymorphic Information Content (PIC) [31].

The pair-wise genetic dissimilarities between individuals were calculated by the Dice algorithm [32], whereas the Nei’s formula was applied to evaluate the inter-population differences [33]. The resultant distance matrices were used to construct dendrograms via the Ward method. The principal coordinate analysis (PCoA) was based on estimators of standardised covariance of genetic distances. The intra-accession genetic diversity was determined by the Nei unbiased coefficient (uHe). The complete data set was also subjected to the analysis of molecular variance (AMOVA) to assess molecular variation among and within the oat landraces [34].

The population structure was also estimated using a Bayesian assignment test as implemented in the program STRUCTURE v. 2.3.4 [35]. An admixture model with correlated allele frequencies was employed. The k (number of populations) values were set from 1 to 15, with ten independent runs for each k (10,000 burn-ins and 100,000 iterations). The Structure Harvester [36] was used to determine the true number of populations. The software CLUMPP 1.1 [37] was used to find optimal alignments of independent runs, and the output was used for cluster visualisation.

Based on the passport data, a geographic distance matrix was calculated using the Geographic Distance Matrix Generator [38]. A matrix of absolute values of differences in collection site altitudes was also calculated.

The Mantel test [39] with 10,000 permutations was conducted to compare proximity matrices of the ISSRs and agro-morphological data and the geographic distance. The Generalized Procrustes Analysis (GPA) was used to reduce the scale effects and to obtain a consensus configuration of the analyses. All the above mentioned analyses were performed using the MS Excel 2010, GenAlex 6.5 [33] and XLSTAT Ecology (Addinsoft, Inc., Brooklyn, NY, USA) softwares.

On the basis of the agro-morphological and molecular marker data, a core set was extracted from the whole tested accessions using the advanced M strategy implemented through a modified heuristic algorithm using the PowerCore [40]. The homogeneity was evaluated using the mean difference (MD%), coincidence rate of range (CR%), variance difference (VD%) and variable rate of the coefficient of variance (VR%) [40,41].

The mean Difference (MD %) was estimated using the equation

$$MD\;\left(\%\right)=\frac{1}{m}\sum_{j=1}^{m}\frac{Me-Mc}{Mc}\times 100$$

where *Me* is the mean of the entire collection, *Mc* is the mean of the core collection and *m* is the number of traits.

The coincidence rate (CR %) was estimated as:

$$CR\left(\%\right)=\frac{1}{m}\sum_{j=1}^{m}\frac{Rc}{Re}\times 100$$

where *Re* is the range of the entire collection, *Rc* is the range of the core collection and *m* is the number of traits.

The variance Difference (VD %) was estimated as:

$$VD\;\left(\%\right)=\frac{1}{m}\sum_{j=1}^{m}\frac{Ve-Vc}{Vc}\times 100$$

where *Ve* is the variance of the entire collection, *Vc* is the variance of the core collection and *m* is the number of traits.

The variable rate of the coefficient of variation (VR %) was estimated as:

$$VR\left(\%\right)=\frac{1}{m}\sum_{j=1}^{m}\frac{CV_{c}}{CV_{e}}\times 100$$

where *CV*_*e*_ is the coefficient of variation of the entire collection, *CV*_*c*_ is the coefficient of variation of the core collection and *m* is the number of traits.

The coverage of all agro-morphological traits in the entire collection was estimated in the core as:

$$Coverage\;\left(\%\right)=\frac{1}{m}\sum_{j=1}^{m}\frac{Dc}{De}\times 100$$

where *Dc* is the number of classes occurring in the core subset, *De* is the number of classes occurring in the entire set and *m* is the number of traits [40].

## Results

### Agro-morphological diversity

The agro-morphological data of the Polish common oat landrace collection, assessed in the field trials, was obtained from the EGISET [25] database. The results were available for the majority of the accessions (84 accessions). Scores for the following traits are summarised in Table 2: plant height, heading date, days to maturity, lodging, resistance to powdery mildew and crown rust, thousand seed weight and yield. A wide range of phenotype scores were found for the tested traits, as indicated by generally high values of the H index (Table 2). In general, the tested accessions showed a high resistance to crown rust and powdery mildew, only few were affected by the pathogens in a moderate degree. The grain yield was the trait that showed the highest differentiation among the tested landraces.

**Table 2:** Agro-morphological trait values.

trait	units	Mean	sd	min	max	H	accessions near min	accessions near max
heading date	days	74.8	4.5	60	82	1.94	PL 51439 _(44)_	PL 51612 _(62)_
							PL 51443 _(48)_	PL 50529 _(15)_
							PL 51442 _(47)_	PL 50531 _(17)_
days to maturity	days	116	1.6	111	119.5	1.78	PL 52268 _(80)_	PL 51442 _(47)_
							PL 51687 _(71)_	PL 51440 _(45)_
							PL 51443 _(48)_	PL 52351 _(85)_
plant height	cm	126.3	8.5	100.7	146.9	2.05	PL 51632 _(69)_	PL 50531 _(17)_
							PL 52195 _(76)_	PL 50529 _(15)_
							PL 50530 _(16)_	PL 51522 _(51)_
lodging	scale 1–9	5	1.3	1	8	1.82	PL 50530 _(16)_	PL 51443 _(48)_
							PL 50593 _(23)_	PL 51439 _(44)_
							PL 50613 _(24)_	PL 51522 _(51)_
powdery mildew	scale 1–9 (	7.9	0.6	6	9	0.79	PL 51439 _(44)_	PL 51609 _(59)_
							PL 51441 _(46)_	PL 51632 _(69)_
							PL 51442 _(47)_	PL 51687 _(71)_
crown rust	scale 1–9 (	7.3	0.7	5	8	1.01	PL 52255 _(77)_	PL 52433 _(90)_
							PL 51632 _(69)_	PL 51609 _(59)_
							PL 52193 _(74)_	PL 51687 _(71)_
yield	g	1462	308.5	821.9	2058.9	2.08	PL 52351 _(85)_	PL 52344 _(84)_
							PL 52191 _(73)_	PL 50076 _(1)_
							PL 52193 _(74)_	PL 51600 _(53)_
thousand seed weight	g	28.4	3.1	22.9	35.11	1.99	PL 50381 _(5)_	PL 51615 _(64)_
							PL 51522 _(51)_	PL 52195 _(76)_
							PL 50521 _(12)_	PL 50698 _(30)_

[H—Shannon-Weaver diversity index]

**Fig 1:**
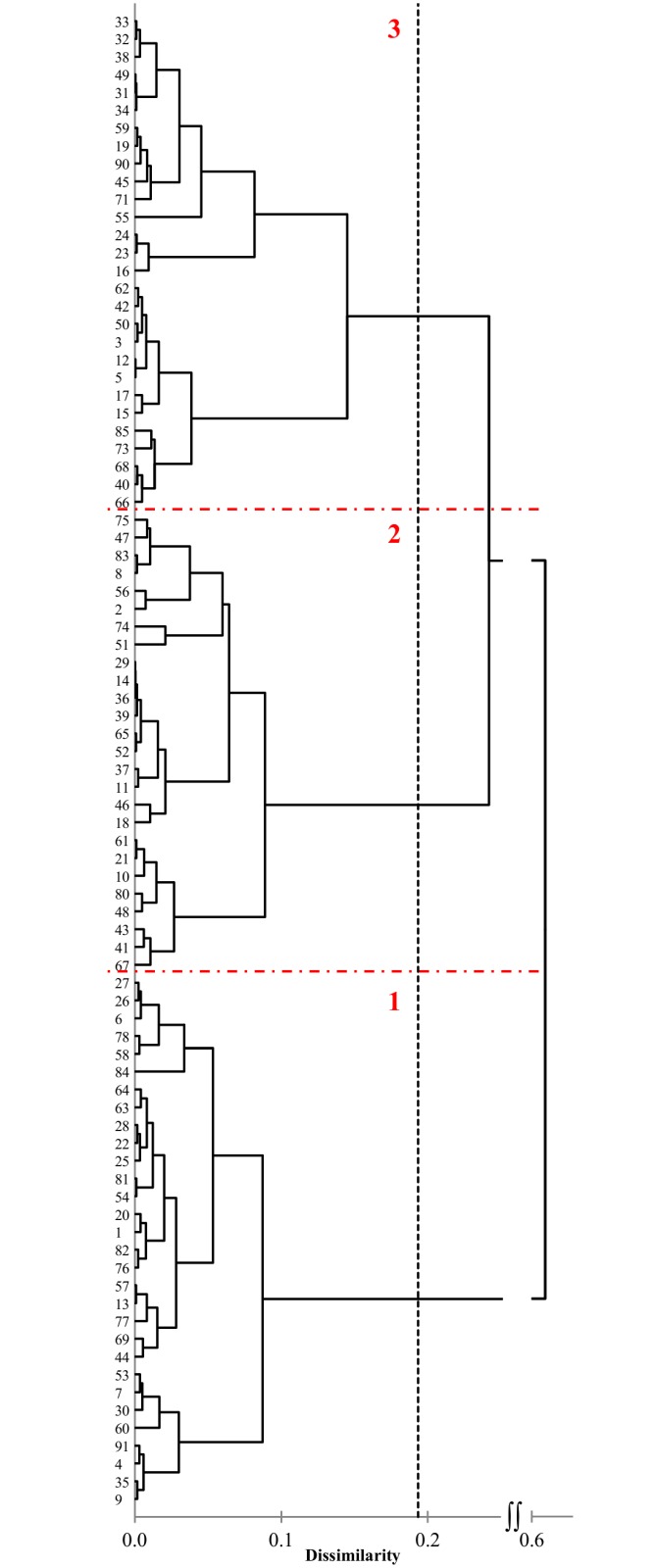
Dendrogram obtained from agro-morphological traits of 84 oat landraces using Ward’s algorithm with three major clusters marked by dashed line.

The similarity measured by the Gower coefficient was in the range of 0.52–0.98. The cluster analysis of 84 accessions identified three distinct clusters composed of 30, 26 and 28 accessions (Fig 1). Within three main clusters the lower levels subdivision was detected. The first cluster included the high yielding landraces, whereas the third one comprised the early accessions with a low thousand seed weights. The landraces placed in the second cluster generally had moderate values of the traits.

**Fig 2:**
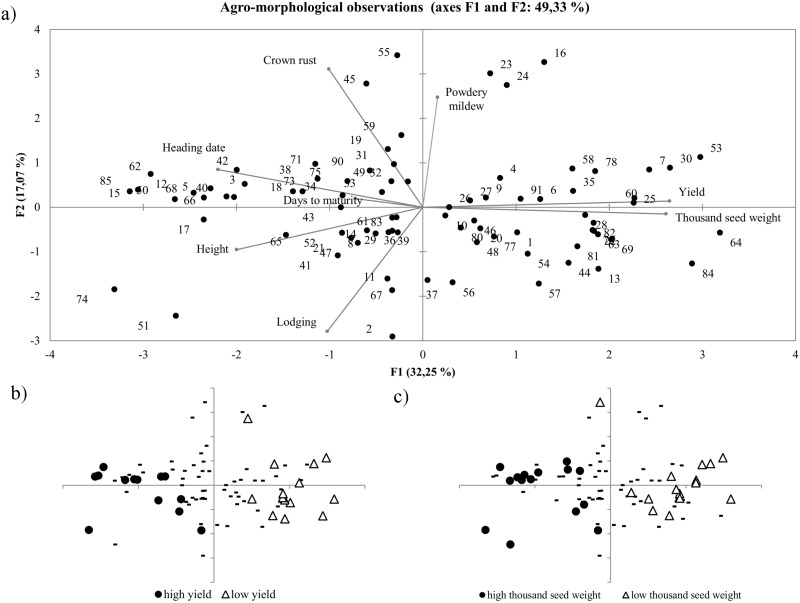
Principal components analysis results based on agro-morphological traits. Bi-plots of the first two principal components F1 and F2. a) PCA including bi-plots based on agro-morphological traits; b) PCA plot with accessions coded by high or low yield; c) PCA plot with accessions coded by high or low thousand seed weight.

In the principal components analysis (PCA), the first three principal components (with eigenvalues > 1) explained 63.56% of the variation. The first axis, explaining 32.3% of the variation, was linked to the variables yield, thousand seed weight (correlation coefficient, r > 0.8) and heading date (r = -0.7). The second axis (17.1% of variation) was moderately correlated with lodging, crown rust resistance and days to maturity (r>|0.5|) (Fig 2). The third axis, explaining 14.2%, was positively correlated with number of days to maturity (r > 0.7). It was difficult to clearly identify separate groups on the bi-plot; only single accessions performed some separateness. Five accessions, PL 50530 _(16)_, PL 50593 _(23)_, PL 50613 _(24)_, PL 51440 _(45)_ and PL 51604 _(55)_, located outside of the group in the first and second quadrant, were highly resistant both to crown rust and powdery mildew. Three distinct accessions from the third and fourth quadrant PL 50079 _(2)_, PL 552344 _(84)_ and PL 51615 _(64)_ had high thousand seed weight values.

Some pairs of the agro-morphological traits were correlated in the tested genotypes (Table 3). The thousand seed weight was significantly correlated with the yield, heading date, plant height and crown rust resistance. The grain yield was also significantly correlated with the heading date, plant height and lodging. In contrast, a weak correlation was noted between the plant height and the lodging, whereas a strong positive correlation was found between yield and thousand seed weight. The high degree of correlation of the thousand seed weight and the yield with other traits was supported by the PCA analysis where both traits were the primary sources of variation in the first PC axis.

**Table 3:** Values of Pearson’s correlation coefficient among the agro-morphological traits.

	heading date	days to maturity	plant height	lodging	powdery mildew	crown rust	yield
days to maturity	0.30	x					
plant height	0.23	*ns*	x				
lodging	*n*	*ns*	0.29	x			
powdery mildew	*ns*	*ns*	*ns*	*ns*	x		
crown rust	*ns*	*ns*	*ns*	*ns*	0.23	x	
yield	-0.46	*ns*	-0.44	-0.31	*ns*	*ns*	x
thousand seed weight	-0.53	*ns*	-0.37	*ns*	*ns*	-0.25	0.66

[p < 0.05; ns—not significant]

### Genetic diversity

**Fig 3:**
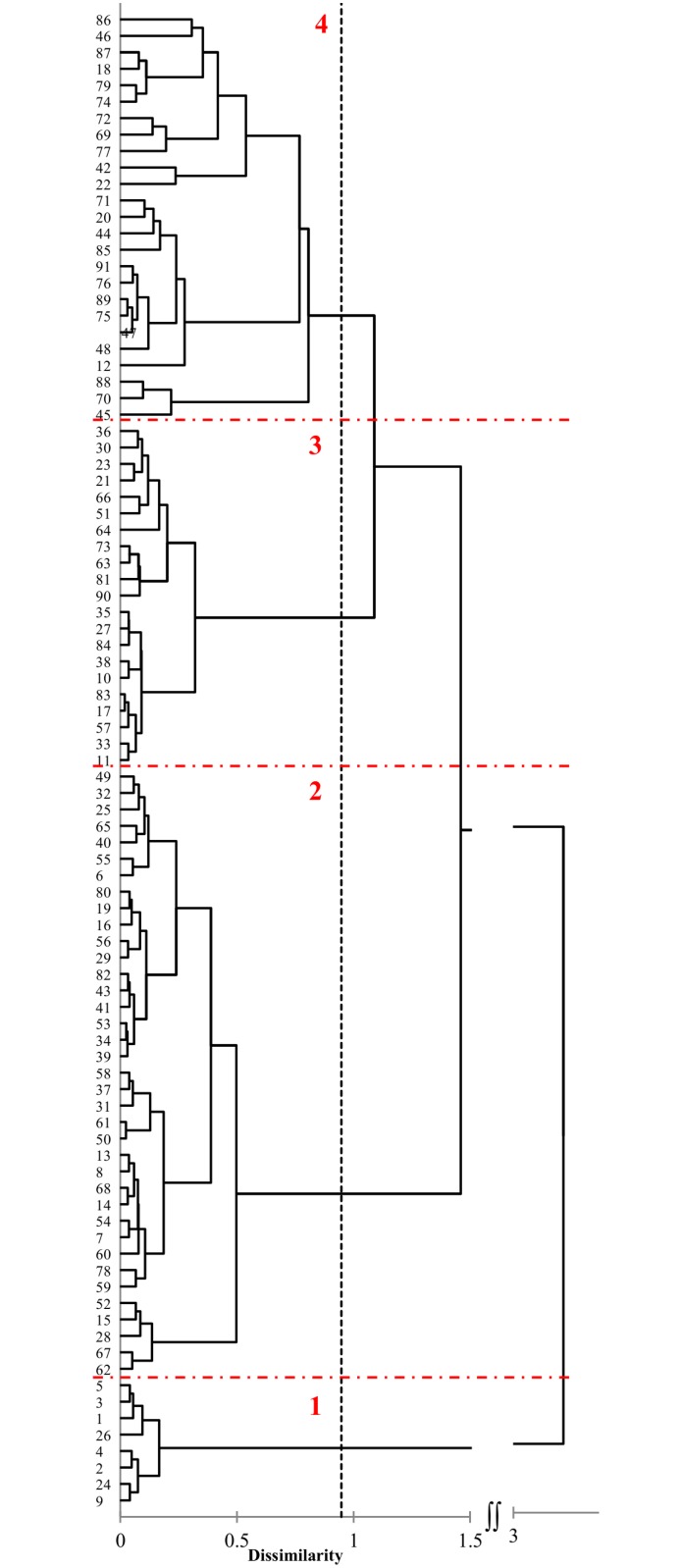
Dendrogram obtained from ISSR data of 91 oat landraces using Ward’s algorithm.

**Fig 4:**
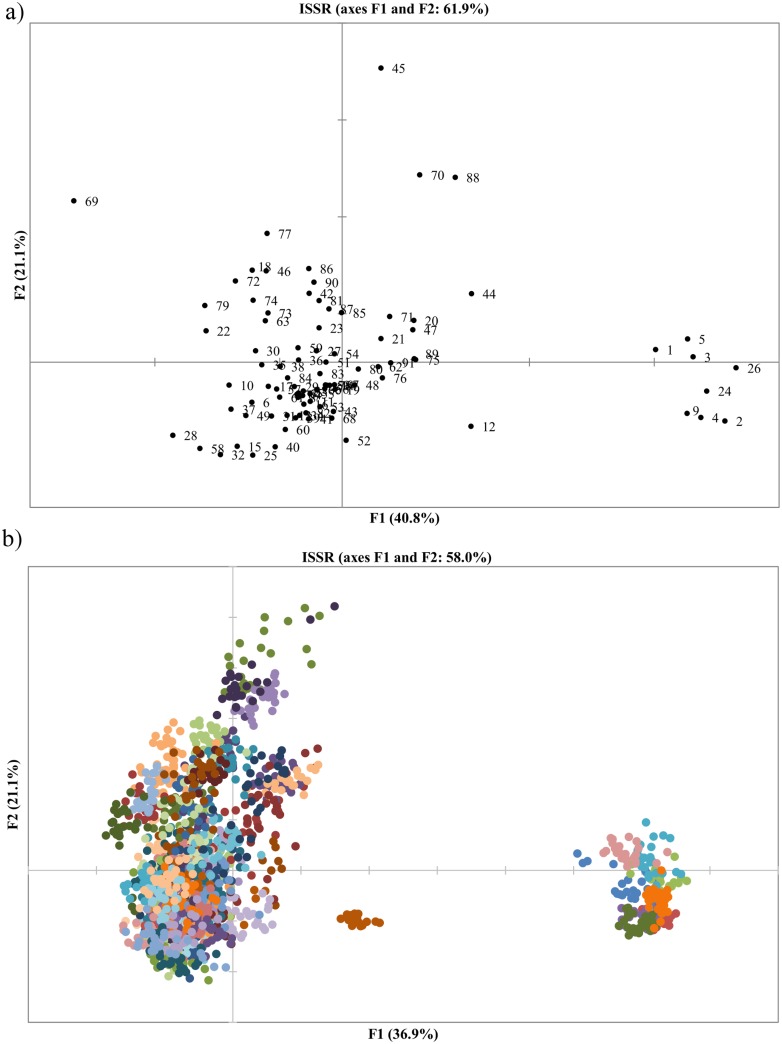
The principal coordinate analysis results based on ISSR data. Bi-plots of the first two principal coordinates F1 and F2. a) based on pair-wise Nei genetic distances among accessions, b) based on Nei genetic distance calculated between all individual plants.

In total, we analysed 1,520 polymorphic loci. Among them, 199 had frequencies lower than 0.05 and were classified as rare. No private alleles were detected. More detailed marker statistics are shown in the Table 4. The polymorphic information content (PIC) varied among the primers from 0.24 (UBC884) to 0.5 (UBC841). A total of 461 fragments (30%) were characterized by high PIC values, i.e. above 0.4, and thus were highly polymorphic. As much as 59% of the fragments amplified by UBC841 had PIC > 0.4. The Nei unbiased genetic diversity (uHe) within landraces ranged from 0.17 PL 52429 _(86)_ to 0.31 PL 51439 _(44)_ (Table 1). AMOVA determined that the majority of the observed genetic variability was due to among-accessions variation (59%, p = 0.001). The variation within the accessions accounted for 41% of the total variation. The pair-wise Nei genetic distances ranged from 0.02 to 0.57. The Ward clustering algorithm based on the Nei distance reflected four main clusters (Fig 3) composed of 8, 37, 21 and 25 accessions. Notably, the fourth cluster contained mainly the landraces of unknown origin, while accessions from the South and the South-East Poland regions constituted the core of the second cluster. Based on the Nei genetic distance calculated between all the individual plants, the principal coordinate analysis was performed. The scatter-plot of the first two coordinates is shown in the Fig 4a, and two major groups were clearly identified. The first, smaller group was located in the first and fourth quadrant and consisted of eight accessions PL 50076 _(1)_, PL 50079 _(2)_, PL 50338 _(3)_, PL 50345 _(4)_, PL 50381 _(5)_, PL 50503 _(9)_, PL 50613 _(24)_ and PL 50622 _(26)_. Three accessions classified earlier as the obsolete varieties i.e. PL 50076 _(1)_, PL 50079 _(2)_ and PL 50381 _(5)_ were located in this group. It allows to suppose, that remaining five accessions might be also erroneously classified as a landrace instead of being obsolete varieties. The second group was located in the second and third quadrant of the coordinate system and consisted of the remaining 83 landraces. However, six landraces of this group showed some distinctiveness i.e. PL 51632 _(69)_, PL 51440 _(45)_, PL 51634 _(70)_, PL 52431 _(88)_, PL 51439 _(44)_ and PL 50521 _(12)_. The detailed plot clearly shows that the gene pools of individual accessions in both groups overlap (Fig 4b). AMOVA performed for two identified groups showed that the between-groups variance component was 15% and therefore much lower than the within- and among-landraces components (36% and 49%, respectively).

**Table 4:** ISSR Primers statistics.

Primer	Sequence	PIC	Mean no. loci/landrace	Rare fragments
UBC 807	(AG)8T	0.49	106.0124	3
UBC 825	(AC)8T	0.31	37.05815	29
UBC 834	(AG)8YT	0.47	70.46429	0
UBC 841	(GA)8YC	0.50	94.5174	5
UBC 856	(AC)8YA	0.28	31.74222	51
UBC 857	(AC)8YG	0.32	37.81136	9
UBC 884	HBH(AG)7	0.24	26.35943	62
UBC 885	BHB(GA)7	0.43	60.63004	40

[PIC—polymorphic information content]

### Population structure

**Fig 5:**
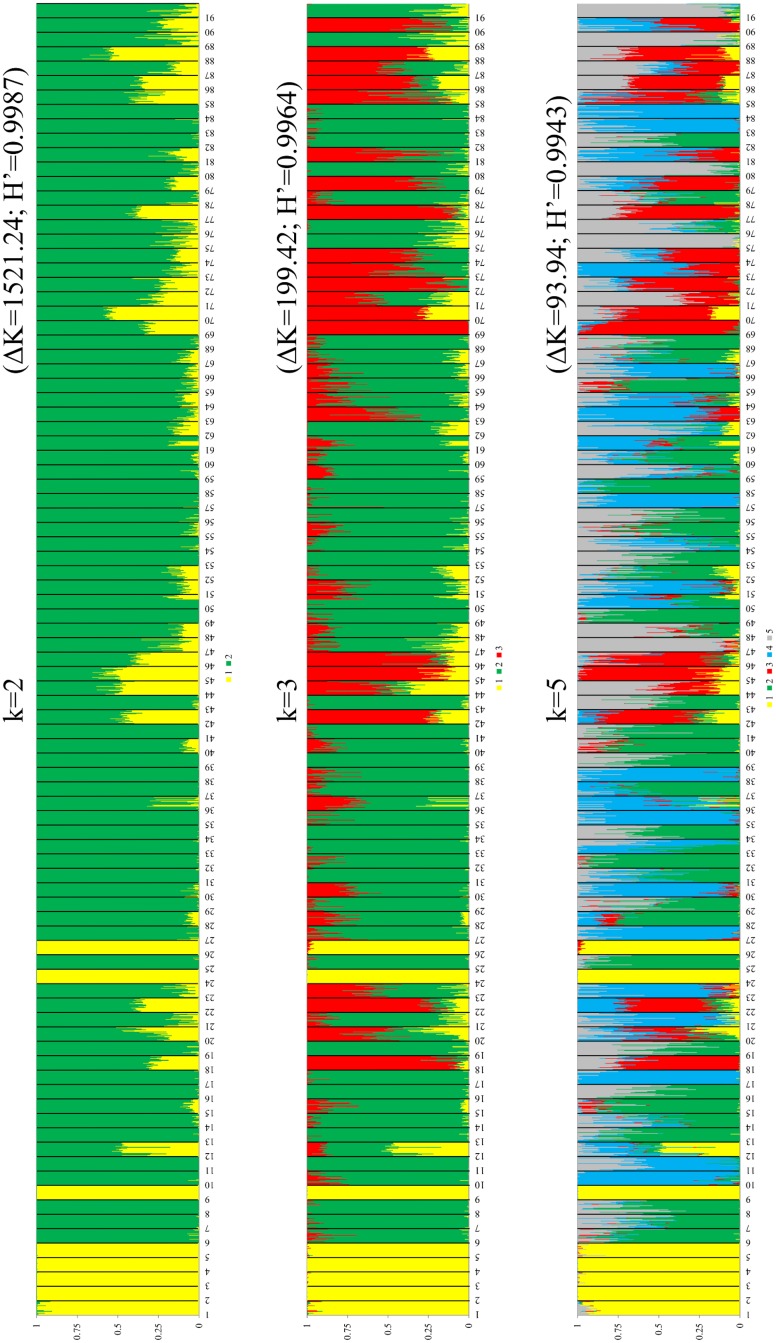
Inference of the population structure of *Avena sativa* landrace collection based on ISSRs using a mode-based Bayesian clustering carried out using the Structure software [35]. The plots were generated based on the Q-matrix consensus permuted across ten replications for k = 2, 3 and 5, using the CLUMPP software [37]. The H’ value indicates the similarity coefficient between ten runs of each k. Each vertical thin bar represents an individual within an accession that is marked by a black border and number. Each number represents a single accession and is consistent with the numbers in Table 1. The assignment of each individual to different gene pools is shown for k = 2, 3 and 5.

**Fig 6:**
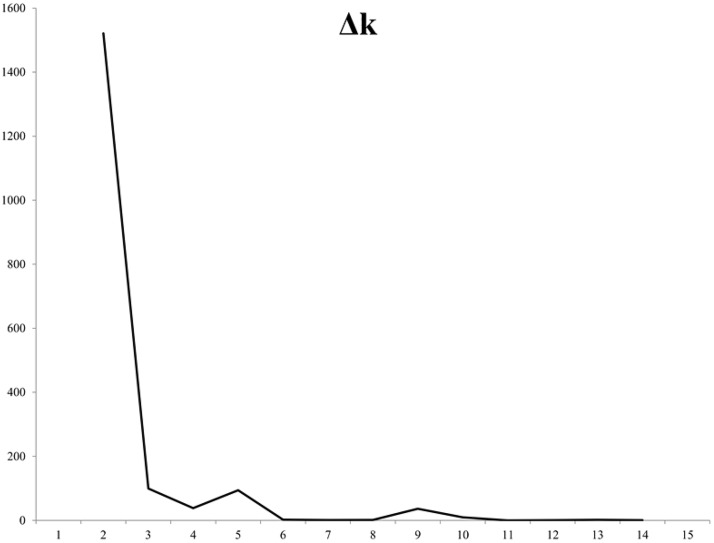
Results of the optimal subpopulation model investigation by plotting ΔK of the data over ten runs, as implemented in Structure Harvester [36].

The STRUCTURE software was run for k = 1–15, based on the distribution of 1,520 different ISSR loci among 2,184 individuals of 91 oat accessions The results of the Bayesian STRUCTURE analysis identifying genetic groupings are illustrated in the Fig 5. The Structure Harvester [36] plotted the Δ against the k numbers of the sub-groups (Fig 6). The maximum ΔK occurred at k = 2 and in the further order, although it was much lower for k = 3 and k = 5. When considering k = 2, the collection was split into two sub-groups (group1, group 2) containing 192 and 1 551 individuals, respectively, while 441 were admixed. The individual was defined as admixed if had less than 80% contribution from one group. The majority of the admixed individuals originated from 17 accessions. Notably, eight accessions of the first group were not admixed at all, irrespective of the predefined k. The much lower probability level of allocation into three or five groups indicated the existence of an internal structure within the initial second group. In addition, a clear differentiation of individuals within the accessions was noticeable when k = 5. Moreover, a significant admixture at this level indicated overlapping in the genetic pool of the oat landraces.

### Comparison and combination of molecular and agro-morphological data

The agro-morphological characterisation was available only for 84 landraces; therefore, the correlation between these traits and ISSR marker system was analysed with the accessions supplied with both sets of data only. The same procedure was used for geographic data. No significant linear relationships were detected between any of the tested pairs of proximity matrices (Table 5).

**Table 5:** Results of the Mantel test.

	ISSR	Agro-morph. traits
Agro-morph. traits	*Ns*	x
Geographic distance	*Ns*	0.047

[p < 0.05; ns—not significant]

A combination of the ISSR and agro-morphological data was analysed using the generalized procrustes analysis (GPA). The consensus proportion for this data was 0.665, but the quantile (78.00) was below the confidence interval (95%).

The manual analysis of the molecular and the agro-morphological data allowed identifying five accessions with a high potential for breeding. The criteria were applied as follows: a high resistance to the pathogens combined with a high yielding and genetic distinctiveness. The selected accessions were: PL 50076 _(1)_, PL 50503 _(9)_, PL 50673 _(28)_, PL 51632 _(69)_ and PL 51439 _(44)_, and it should be noted that one of them had short straw.

### Core collection

The PowerCore selected 21 out of 91 oat landraces, thus reducing the number of accessions by 76.9% for the entire set (Table 1). The mean difference (MD%) exhibits the difference in averages of accessions between the core subset and the entire collection. The low MD% value (5.73%) shows that the mean of the core collection is similar to the mean of the entire collection.

The variance difference (VD%) indicates the difference in the coefficient of variation values existing in the core collection and in the entire collection. The VD% value (30.09%) showed that the variance of the core collection is slightly different from the variance for the entire collection.

The variable rate of the coefficient of variance (VR%), enabling a comparison between the coefficient of variation values existing in the core subset and the entire collection, determines also the level of representation of the starting collection in the core subset. The core had a VR% values of 121.33% over the entire set, indicating a good representation.

The coincidence rate of range (CR%) indicates whether the distribution ranges of each variable in the core set are well represented when compared to the entire set. The resulting CR% value (99.05%) indicated homogenous distribution ranges of agro-morphological traits. The value of calculated coverage coefficient for the resulting core was 100%. This indicates full coverage of the agro-morphological diversity present in the starting collection.

## Discussion

### Genetic diversity

The genetic diversity and the population structure of Polish oat germplasm has been analysed in the past using molecular markers [24,27,28,42,43]. Despite that, little is known about the amount and distribution of the genetic diversity and the population structure within the Polish oat landrace collection, as all above mentioned studies included only fragments of the collection. However, knowledge on the genetic structure within the entire set of landraces is crucial for the applications in breeding and science. In this study, the genetic diversity among and within accessions of 91 oat landraces from Poland was analysed for the first time.

Analysis of the genetic diversity parameters showed a higher level of variability among the indigenous oat landraces than previously reported for 67 oat landraces [27]. Notably, two separate groups of the accessions were identified. A similar situation was observed in our previous study in which, beside landraces, also some varieties were analysed [24]. It is noteworthy that in both of oat diversity studies the same set of ISSR markers was used. In the previous study [24], three landraces were identified as old Polish varieties that had been erroneously classified. These three accessions were also used in this study, allowing us to identify additional five erroneously catalogued accessions which formed the distinct group. Removal of these eight accessions obviously lowered the general diversity of landraces which, surprisingly, was only slightly higher than the diversity of the 19 landraces that were included in the previous study [24]. This indicates that the Polish common oat landraces gene pool is much narrower than expected; therefore, it is reasonable to create a core collection consisting of 21 accessions reflecting the variability of the entire set, that could be used as the active collection. Based on these results, an advantage of landraces over contemporary varieties in terms of gene pool broadness is indisputable. A lack of modern oat varieties diversity has been previously reported [11,44,45]. In the tested accessions we did not found any links between genetic and morphological variation. A similar result was obtained in the study of Winkler et al. [46].

The determination of the internal diversity level of each indigenous landrace was one of the goals set for this study. This type of data is currently not available in the literature because the internal variation was investigated only a few times, although landraces are regarded as heterogeneous [47]. Apart from our previous analysis [24], it appears that the internal variation of oat accessions has been investigated only once. Eight Swedish landraces demonstrated a high degree of internal variation based on SSR data [48]. In our study, the level of the internal diversity of oat landraces is much higher than in the previously tested oat population varieties [24]. The ISSR markers showed the various levels of within-accessions variability. A thorough analysis of the internal diversity reported the absence of significant linkage with the location of collecting sites or the collecting years. In the past the diversity of the oat germplasm collection was investigated several times [16,44,49]. Those studies based on bulk samples, so the internal diversity of oat accessions was not evaluated. In the most recent study performed on 1 000 oat accession from NSGC, DNA from only one plantlet per accession was analysed by iSelect 6K beaded chip array [46]. The authors identified as much as 324 lines as duplicates with one or many other lines. Based on the result of our study we could speculate that the duplicates are a direct derivative of the gene pool overlapping. We could also presume that a single plant represents the gene pool worse than bulk sample.

Currently, we cannot determine precisely enough whether the variation level is specific to the accession or it is a derivative of the size of the collected seeds sample as well the way in which it represented the initial diversity. Without any prior information about the initial variability, we are not also able to prove that the reproduction and regeneration standards of the gene bank were sufficient to maintain the collected diversity. However, we consider that the obtained results can be a valuable reference point for future studies. A DNA biobank established during this study, in the future, will allow researchers a direct comparison of variation level even if they will use different analytical methods.

### Agro-morphological diversity

The goal of our study was also to describe the variability patterns of important agro-morphological traits in the landrace collection in order to improve breeding selection of cross parents. The studies gave also the opportunity to distinguish some germplasm sources for breaking difficult correlations among the characters. Based on data obtained from the gene bank database EGISET [25], the diversity of the accessions was estimated. The whole set of data was completed only for 92% of the collection.

The yield is an extremely complex characteristic, and its components are strongly influenced by a high number of environmental factors. Moreover, its inheritance is neither simple nor favourable for breeding practice. The importance of genetic dominance and non-additive interactions of epistatic genes for yield heritability is significantly greater than the effect of additive genes [50,51]. In more recent oat varieties, the participation of additive direct variance for yield inheritance has increased [52,53]. Generally, landraces are relatively stable-yielding in successive seasons even in less favourable conditions, which is assumed as related to their high internal genotypic diversity. Among the surveyed accessions, we found both very low yielding ones as well as those with yields comparable to modern varieties. However, it should be considered that relatively high initial internal variation and a low to moderate heritability may impede the use of oat landraces in breeding for yield increase, and a long pre-breeding period with low selection pressure may be required.

The plant height is also a very complex character determined by interaction of numerous exo- and endogenous factors. Earlier studies have confirmed the polygenic nature of this trait [54]. No typical dwarfs were present in the studied landrace collection; however, differentiation was remarkable. Some landraces with shorter straw (approximately 100 cm) i.e. PL 51632 _(69)_, PL 52195 _(76)_ and PL 50530 _(16)_ may be useful in the improvement of harvest index and resistance to lodging, in spite of the widespread opinion that landraces considered as cross components are generally too tall.

The analysed accessions were characterised by diverse resistance to lodging; moreover, this characteristic demonstrated variability between years during the field trial. This is related to the complexity of the feature that, similar to plant height, is affected by a high number of endogenous and exogenous factors. Lodging is significantly influenced by environmental conditions such as presence and strength of wind, humidity of the soil surface, fertilisation and the sowing rate. These factors interact with the genetically based characteristics such as plant height, size and properties of tissue mechanical of the lower internodes, straw flexibility, resistance to culm base diseases (*Fusarium avenaceum*, *F*. *culmorum* oraz *Bipolaris sorokiniana*) and development of adventitious roots. Genetic studies of lodging are difficult and rarely performed because of their complexity [55]. The studied landraces are expected to have a level of genetic individuality, shaped as a compensation of tall stature, which was not necessarily linked with increased susceptibility to lodging as in the accessions. PL 51618 _(67)_, PL 52268 _(80)_ and PL 51443 _(48)_.

The length of the vegetation period was among the analysed traits. It was measured as the heading date and days to maturity, both influenced by the seasonal changes of precipitation, temperature and the connected date of sowing. The differences in the heading date were larger than in the days to maturity, which is in agreement with similar studies [55]. The longer photoperiod promotes the earlier flowering, and this response tends to be greater in varieties from higher latitudes [56]. Reducing the length of the vegetation period with simultaneous increase of yield is one of the main breeding goals. Due to a relatively high heritability of the heading date, selection in early generations should be effective. A precise determination of the number of days to maturity is more difficult than determination of the heading date, but with only a little lesser degree of heritability [57,58]. Among the investigated landraces, the accessions PL 52434 _(91)_, PL 51632 _(69)_ and PL 521955 _(76)_ have shown interesting combination of earliness and yield.

Crown rust, caused by *Puccinia coronata* Cda. f.sp. *avenae* Erikss., is one of the most dangerous and the most widely disseminated oats fungal diseases, with the yield losses as high as 40% [55]. The total (vertical) resistance to a pathogen race is a dominant trait determined usually by one or a few dominant genes with high heritability [59,60]. *Puccinia coronata* has the ability to adapt quickly to resistant lines or varieties of oat; therefore, constant search for a new source of resistance is necessary to effectively combat this disease. The investigated set of accessions was characterised by the sufficient resistance to crown rust; however, it was moderately infected. Resistance of heterogeneous landrace populations may be similar to that described in the oat multiline cultivars, in which interaction of polymorphic genes from different individuals caused significantly higher level of resistance than expected basing on the expression in genetically homogenous stocks [61]. Nevertheless, the landraces are still expected to be a good reservoir of resistance mechanisms other than in modern varieties.

Powdery mildew caused by *Blumeria graminis* (DC.) E.O. Speer f.sp. *avenae* Em. Marchal is a serious foliar disease of oats. It is widespread in the north-western Europe and in the south-eastern parts of the US [62,63] and reaches the epidemic levels mostly late in the season in wet summers [64]. The grain yield losses may reach up to 39% in years with high disease pressure [65,66]. Based on literature data, resistance to powdery mildew does not seem to be widespread in *A*. *sativa* [67]. Similarly, to crown rust, the analysed accessions showed low infection levels, but this some doubt, that might have been caused partly by weather conditions unfavourable to the pathogen and low disease pressure, not by plant resistance.

The thousand seed weight (or thousand kernels weight—TKW) is a relatively stable factor for oat variety characterisation; however, it may be significantly decreased by biotic or abiotic stress at the end of the vegetation period. However, this trait is considered by breeders as much less important compared to percentage of husk or kernel size uniformity, which were not included in this study.

### Core collection

The NCPGR preserves about 80 000 accessions of different species. All of them are available as an active collection. Regeneration of such amount of accessions is difficult and expensive, therefore it is reasonable to create core collections reflecting the variability of the preserved genus or species for common use as an active collection. The core collection of common oat landraces is the first one in NCPGR. It represents the genetic and morphological diversity of the entire landrace collection. Besides the gene bank benefits, the smaller set of well described accessions may act as incentive for breeders to incorporate obsolete germplasm into the breeding programs.

Oat landraces have a considerable potential for use in improving disease and abiotic stress tolerance. Additionally, compared to the use of crop wild relatives, there are no obstacles to transfer the valuable characteristics from landraces to new varieties. However, it needs to be remembered about the high level of internal variability that may complicate the process of breeding. The oat landraces provide a wide spectrum of the starting material for breeding programs, as they make it possible to choose the traits which are best suited to local conditions. They are adapted to local and show resistance to local diseases, with an increased ability to compete with indigenous weeds. Despite the large height diversification of the oat plants observed in this study, they tended to be high, which constitutes an indispensable characteristic for weed suppression. Therefore, it has to be concluded that oat landraces should also be grown and preserved *in situ*. This, however, requires both scientific expertise and traditional knowledge of farmers who should grow and manage oat landraces in places where the population originated.

Our results show the high genetic diversity of Polish common oat landraces. However, the broadness of the gene pool turned out to be lower than previously expected, but was still significantly higher than that of contemporary varieties. Compilation of molecular and agro-morphological data allowed to select a set of landraces which may be a valuable “new “recommended source of diversity for breeding programs. Moreover, based on the presented results, we can conclude that many of the landraces which are preserved in the Polish gene bank may be directed to the *in situ* conservation, which may be satisfactory for both farmers and scientists.
